# Factors associated with physician modifications to automated ECG interpretations

**DOI:** 10.1093/ehjdh/ztaf119

**Published:** 2025-11-08

**Authors:** I Min Chiu, Yuki Sahashi, Sam S Torbati, Sumeet S Chugh, David Ouyang

**Affiliations:** Department of Cardiology, Smidt Heart Institute, Cedars-Sinai Medical Center, Los Angeles, CA, USA; Department of Cardiology, Smidt Heart Institute, Cedars-Sinai Medical Center, Los Angeles, CA, USA; Department of Cardiology, Gifu University, Gifu, Japan; Department of Emergency Medicine, Cedars-Sinai Medical Center, Los Angeles, CA, USA; Department of Cardiology, Smidt Heart Institute, Cedars-Sinai Medical Center, Los Angeles, CA, USA; Department of Cardiology, Smidt Heart Institute, Cedars-Sinai Medical Center, Los Angeles, CA, USA; Department of Cardiology, Kaiser Permanente Santa Clara Medical Center, Santa Clara, CA, USA

**Keywords:** 12-Lead electrophysiology, Automated interpretations, Physician modifications

## Abstract

**Aims:**

Accurate diagnoses contribute to the improvement of clinical workflows and the enhancement of patient care. Commercially available automated electrocardiogram (ECG) interpretation systems require manual review by physicians despite their widespread use. This study investigates the frequency and characteristics of the modifications from automated ECG reports in routine clinical practice.

**Methods and results:**

We retrospectively analysed 159 630 ECGs from 2011 to 2023 and compared automated preliminary ECG reports generated by the GE Marquette™ 12SL ECG analysis programme with finalized reports by physicians. A modification was defined as any textual difference between the initial and final reports. Our analysis revealed that 31.3% of all ECG reports underwent some forms of modification by physicians. We analysed the frequency of 69 pre-defined ECG-related terms before and after physician review, categorizing modifications as unchanged, deleted, or newly added. Modifications were more frequent for ECGs performed during off-hours, in patients with higher ventricular rates and longer QRS durations. At the term-level, diagnoses such as ‘prolonged QT interval’ (newly added from 5.6% of original reports) and ‘electronic ventricular pacemaker’ (newly added from 3.6% of original reports) were frequently added by physicians, while diagnoses like ‘inferior infarct’ and ‘anterior infarct’ were frequently deleted from automated ECG reports (32.0% and 44.6% automated reports with these terms required removals).

**Conclusion:**

This large-scale real-world study demonstrated the high frequency of physicians’ modification in automated ECG interpretation. The identified patterns of modifications highlight the limitations of current rule-based systems in handling complex cases and nuanced ECG findings.

## Graphical Abstract

**Graphical Abstract:**
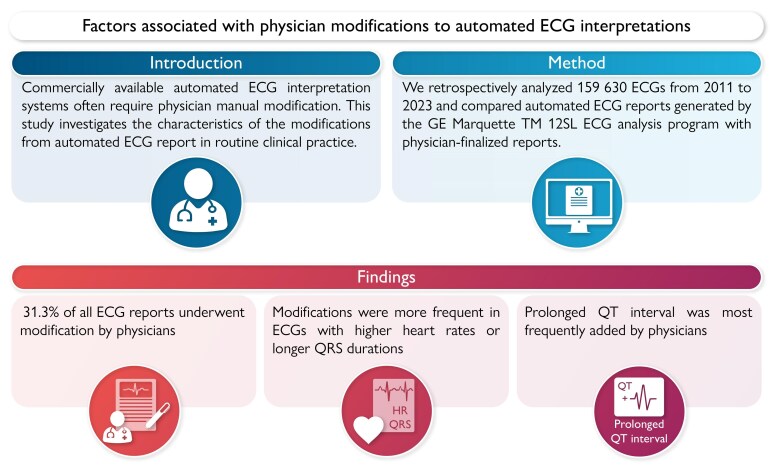

## Introduction

Electrocardiography (ECG) is among the most routinely performed diagnostic tools in clinical practice, widely used for the early detection of cardiovascular diseases. Millions of ECGs are recorded annually worldwide, with most automatically analysed by algorithms that generate real-time preliminary interpretations to support clinical decision-making, streamline workflow, and reduce human error.^1^ Several commercial automated ECG interpretation systems have been widely adopted in clinical settings.^2,3^ Despite substantial advancements and widespread adoption, automated ECG interpretations are not yet perfectly accurate, frequently requiring physician modification to ensure clinical diagnostic accuracy. Prior studies have highlighted various issues with these automated systems, including misinterpretation of subtle or complex ECG findings, over-reliance on standardized patterns, and poor generalization to diverse patient populations.^4,5^ These inaccuracies potentially lead to incorrect clinical management, unnecessary diagnostic testing, or failure to recognize critical conditions, thereby negatively impacting patient safety and healthcare resource utilization.

Despite the reliance on automated ECG interpretation, there is a limited comprehensive understanding of the real-world interaction between these automated algorithms and expert modification. Prior research predominantly focused on validating algorithmic accuracy against expert consensus, often under controlled conditions, leaving real-world clinical applicability relatively unexplored.^6^ Furthermore, a previous study examined narrow diagnostic subsets or limited patient demographics, which inadequately represent diverse clinical practice scenarios.^7^ As automated ECG interpretations become increasingly integrated into daily clinical practice, it becomes essential to systematically analyse the patterns and frequencies of modifications physicians made. Identifying common patterns and understanding the clinical contexts that trigger modifications can guide improvements in algorithms. This understanding is particularly crucial in high-risk clinical scenarios where prompt and accurate ECG interpretation directly influences patient outcomes. Mitigating these gaps necessitates a detailed analysis of a large and diverse set of ECG reports to systematically identify specific ECG features and clinical scenarios frequently misinterpreted or overlooked by automated systems.

The objective of this study is to comprehensively examine the frequency, characteristics, and context of modifications physicians made to automated ECG interpretations in routine clinical practice. Specifically, we aim to identify the particular ECG findings most often modified by clinicians, analyse the patterns of these modifications and evaluate the relationship between physician modification rates and patient demographics, clinical conditions, and temporal factors.

## Methods

### Data source and study population

We retrospectively collected all 12-lead ECGs at Cedars-Sinai Medical Center (CSMC) between 1 January 2011, and 31 December 2023. All ECGs were acquired at rest using a GE Healthcare ECG recording machine, and the corresponding automated interpretations generated by the GE Marquette™ 12SL ECG analysis^8^ programme, and their corresponding analysis programme version were also obtained. All 12-lead ECG recordings were acquired in outpatient clinics, inpatient wards, surgery rooms, and the emergency department. During the modification process, all ECG corrections were made via free-text entry. From all ECGs, we excluded reports not reviewed by physicians. To adjust for the skewness of the modification rate by physicians, we further excluded ECG reports reviewed by physicians who were outliers with a very high rate of changes (e.g. physicians who made some alteration in over 95% of their ECG reports). In addition, given the highly skewed distribution of the number of ECGs interpreted per physician, with a small number of physicians reading the vast majority of all ECG readings (see Supplementary material online, *Figure S1*-*SA*), we randomly sampled a total of 80 000 ECGs from eight physicians (10 000 ECGs from each) whose interpretation numbers were more than three standard deviations above the mean (see Supplementary material online, *Figure S1*-*SB*).^9^ The study flowchart is described in Supplementary material online, *Figure S2*. For each ECG, the automated report generated by the GE Marquette™ 12SL system and the reports finalized by the physician were compared.

### Analysis of term frequency and modifications

A ‘Modified report’ was defined as any case where there was a textual difference between the initial automated report and the reports reviewed by physicians. In contrast, cases where the physician finalized the report without any textual alteration were categorized as an ‘Unchanged report’. To systematically analyse the modification patterns made to the automated ECG reports, we identified a comprehensive list of 69 unique and exclusive words commonly used and associated with ECG findings and diagnoses. These terms were independently reviewed by two physicians (I.M.C. and Y.S.) to cover a broad spectrum of ECG findings and diagnoses. Based on their clinical relevance and similarity, the remaining 69 terms were further categorized into 9 distinct categories (e.g. ‘Pre-mature beats’, ‘Conduction disorders’, ‘Ischaemia related’, ‘Cardiac hypertrophy/enlargement’, etc.). The list of all specific 69 ECG terms and their corresponding 9 categories are summarized in Supplementary material online, *Table S1*. We calculated the proportion of each ECG term included in the unchanged report group and the modified report group. Within the modified report group, we analysed how often the selected ECG-related terms changed before and after physician review. For each ECG term, we calculated the following metrics:

1. Term frequency in the original report: The total number of times each ECG term appeared in the automated reports before physician review.
2. Original report count: The number of individual automated original reports in which the ECG term was present in the automated report before physician review.
3. Term frequency after physician review: The total number of times the ECG term appeared in the reports reviewed by physicians after review.
4. Final report count: The number of individual reports in which the ECG term was present in the reports reviewed by physicians after review.

**Figure 1:**
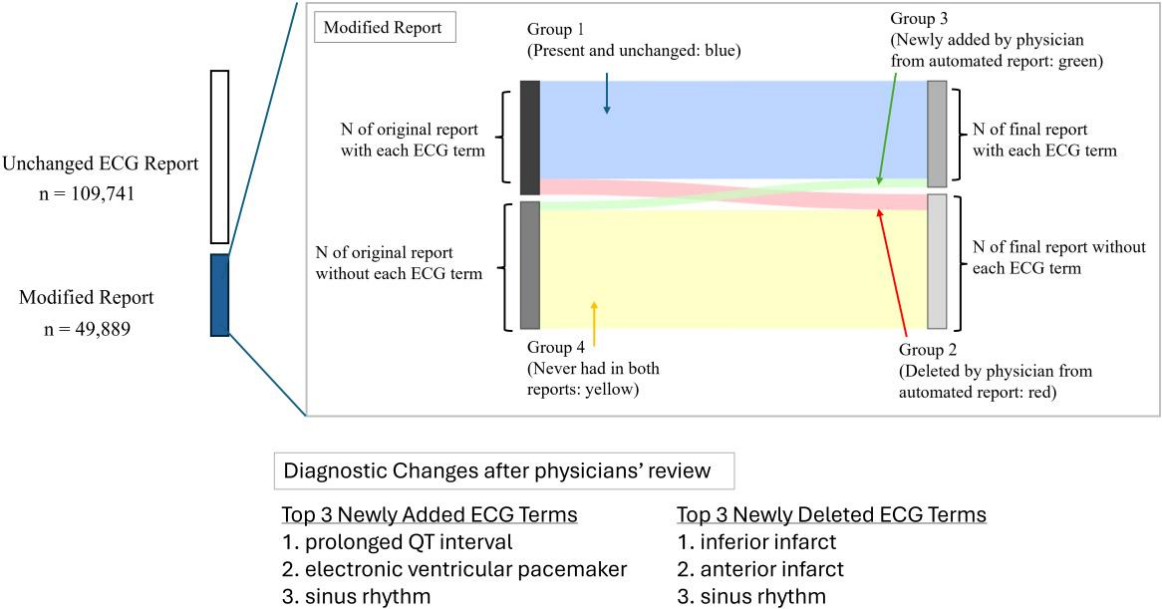
Study overview. Pattern of ECG term modifications following physician review. Group 1 (blue) shows terms present in both automated and final reports. Group 2 (red) represents terms deleted by physicians’ review from automated ECG report. Group 3 (green) indicates terms newly added by physicians from the original report that did not contain that ECG term. Group 4 (yellow) represents terms absent in both automated and reviewed reports. Arrows and connecting bands visualize the transition of ECG terms, with the band thickness proportional to the number of reports.

To understand the nature of the modifications at the individual ECG term-level, we categorized each automated report based on the ECG term presence before and after physician review (*Figure 1*):

- Group 1 (Unchanged): The term was present in the automated report and remained in the reports reviewed by physicians.
- Group 2 (Deleted from automated reports by physician): The ECG term was present in the automated report but was removed in the reports reviewed by physicians.
- Group 3 (Newly added from automated reports by physician): The ECG term was absent in the automated report but was newly added by physicians to the final reports.
- Group 4 (Never appeared in both reports): The ECG term was absent in both the automated report and the reports reviewed by physicians.

We then calculated the following metrics for each ECG term:

- Added report ratio: Calculated as the number of times a term was added (Group 3) divided by the total number of times the term was absent in the automated reports (Group 3 + Group 4) for each ECG term.
- Deleted report ratio: Calculated as the number of times a term was deleted (Group 2) divided by the total number of times the term was initially present in the automated reports (Group 1 + Group 2) for each ECG term.

The collected data, including the modification patterns for each of the ECG terms, were further analysed based on arrhythmia categories, physician experience, and acquisition year. For continuous variables, a *t*-test was used to compare two groups, and for categorical variables, a χ^2^ test was used. Data analysis was performed using both Python (version 3.10.12) and R (version 4.2.2) programming languages. This study received approval from the Institutional Review Board of CSMC.

## Results

During the study period between 1 January 2011, to 31 December 2023, a total of 769 088 resting ECGs were performed and of which 159 630 were finalized by a total of 104 physicians (mean post-graduate year: 24.3 ± 12.9) and were included in the final analysis cohort. In this cohort, the median number of ECGs reviewed by each physician was 177 (inter-quartile range [IQR] 87–994). Of these finalized reports, 49 889 (31.3%) underwent modifications between the initial automated reports and the final physician assessments. More recent ECGs were processed using updated version of the Marquette™ 12SL ECG analysis programme, showing a substantial decline in physician modification rate (42.2% in 2011–12 to 25.6% in 2021–22 and 18.4% in 2023, *P* for trend < 0.001) (*Table 1*). The modification rate by department where ECG were performed is listed in Supplementary material online, *Table S2*.

**Table 1:** Yearly distribution of ECG modification rates and analysis programme utilization

			Marquette™ 12SL ECG analysis programme						
Period	Modification rate	Total count	Version 16	Version 18	Version 19	Version 20	Version 21	Version 22	Version 23
2011/2012	42.23%	49 185	217	44	5019	30 672	13 230	3	0
2013/2014	30.86%	13 110	11	9	55	4953	2321	5761	0
2015/2016	47.57%	9600	0	0	1	923	187	8489	0
2017/2018	39.38%	10 838	0	0	0	1710	1370	7758	0
2019/2020	21.72%	11 973	0	0	0	1516	951	7784	1722
2021/2022	25.59%	23 343	0	0	0	5	34	11 330	11 974
2023	18.43%	41 572	0	0	0	15	8	859	40 690

**Figure 2:**
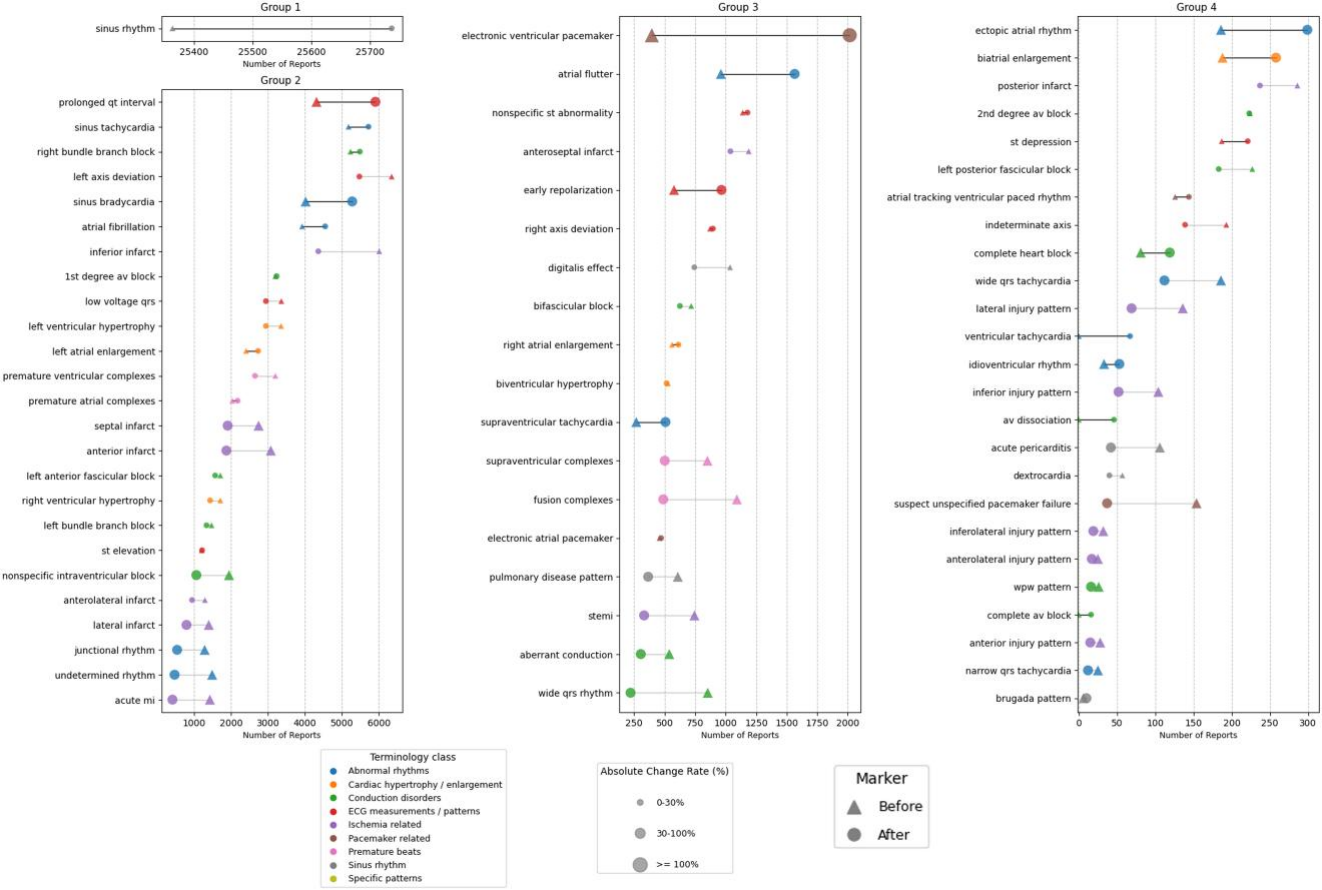
The figure displays the number of reports containing specific ECG terms before and after physician review, categorized into nine ECG terminology groups. The number of reports before review (automated report) are denoted by a triangle and the number of reports after physician review are denoted by a circle. The connecting lines between the two markers indicate the change in the number of reports for each term. A black line demonstrates an increase in the number of reports containing the term after physician review, while a grey line indicates a decrease. The colour of the term label corresponds to the 9-terminology categories as indicated.

Our analysis revealed that report modifications were slightly more frequent for ECGs performed during off-hours (54% vs. 52%, *P* < 0.001) and in patients with a higher ventricular rate (86 ± 28 b.p.m. vs. 80 ± 21 b.p.m., *P* < 0.001) (*Table 2*) and a longer QRS duration (103 ± 33 ms vs. 97 ± 25 ms, *P* < 0.001). For all ECG terms, the percentages included in the unchanged report group and the modified report group were summarized in Supplementary material online, *Table S3*. For each of the 69 ECG-related terms, the number of final reports, which included each term in the modified report group, was described in *Figure 2*. Overall, out of the 69 analysed ECG-related terms, 23 ECG terms showed a net increase in their total count in the reports reviewed by physicians compared with the initial automated reports. (*Figure 2*) The absolute change ratio varied largely across variables from 0.44% (‘2nd degree av block’; pre-report number: 236, post-review report number: 230) to 356.5% (‘electronic ventricular pacemaker’; pre-report number: 446, post-review report number: 2036). We presented a full list demonstrating the total count of the term newly added or deleted, both before and after physician review for each of the 69 ECG-related terms in Supplementary material online, *Table S4*. The table provides a detailed overview of how the presence and frequency of specific ECG findings and diagnoses were changed during the physician review process.

**Table 2:** ECG characteristics among analytic cohort data

Characteristic	Modified Report, *n* = 49 889^a^	Unchanged Report, *n* = 109 741^a^	*P*-value
Age (years)	59 (25)	62 (20)	<0.001
Gender: Female	21 412 (43%)	49 795 (45%)	<0.001
Ventricular rate (b.p.m.)	86 (28)	80 (21)	<0.001
Atrial rate (b.p.m.)	96 (55)	85 (42)	<0.001
QRS duration (ms)	102 (33)	97 (25)	<0.001
QTc (ms)	460 (53)	451 (42)	<0.001
ECG on weekday	40 634 (81%)	90 709 (83%)	<0.001
ECG during off-hours	26 804 (54%)	56 849 (52%)	<0.001

^a^Mean (SD); *n* (%)

**Figure 3:**
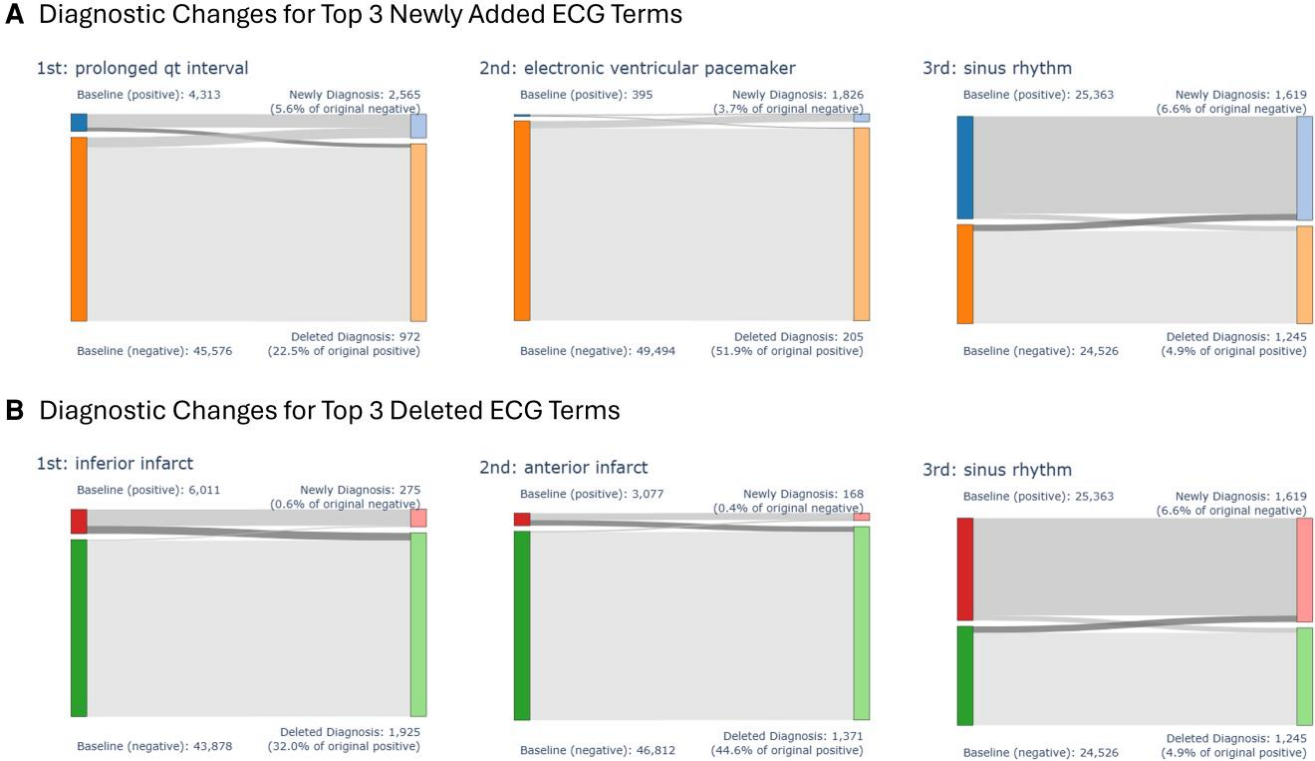
The diagnostic changes associated with the top three newly added (*A*) and top three deleted (*B*) ECG terms after physician review. Each Sankey diagram depicts the flow of reports between the initial automated diagnosis (left side) and the final physician-assigned diagnosis (right side) for a specific ECG term.

Among the 69 ECG-related terms, the term most frequently newly added by physicians was ‘prolonged QT interval’ (number of newly added reports = 2565), followed by ‘electronic ventricular pacemaker’ (*n* = 1826) and ‘sinus rhythm’ (*n* = 1619). Conversely, the term most often deleted from the automated reports by physicians was ‘inferior infarct’ (number of newly deleted reports = 1925), followed by ‘anterior infarct’ (*n* = 1371) and ‘sinus rhythm’ (*n* = 1245). The diagnostic flows of these newly added top 3 ECG terms and deleted top 3 ECG terms are described in *Figure 3*. Certain specific terms exhibited a high deletion rate when present in the automated report. For instance, when the automated report included the term ‘suspect unspecified pacemaker failure’, the term was deleted from 90.3% of reports (*n* = 139) that included this ECG term. Similarly, ‘atrial tracking ventricular paced rhythm’ was deleted from 92.1% of the reports (*n* = 116) where it appeared, and ‘WPW pattern’ was deleted from 84.6% of such cases (*n* = 22). Conversely, ‘prolonged QT interval ‘was added to 5.6% (*n* = 2565) of reports, which were not included in the initial automated report, as well as ‘sinus rhythm’ (6.6%, *n* = 1619) and ‘electronic ventricular pacemaker’ (3.7%, *n* = 1826). (*Figure 3* and Supplementary material online, *Table S4*) Analyses were performed on the top 3 modification patterns of ECG terms by two groups of physicians: the eight physicians from whom ECGs were randomly sampled, and all other physicians. In both groups, ‘prolonged QT interval’ was the most frequently added ECG term. Regarding deleted terms, ‘inferior infarct’ was identified within the top 3 in both groups. (see Supplementary material online, *Table S5*).

**Figure 4:**
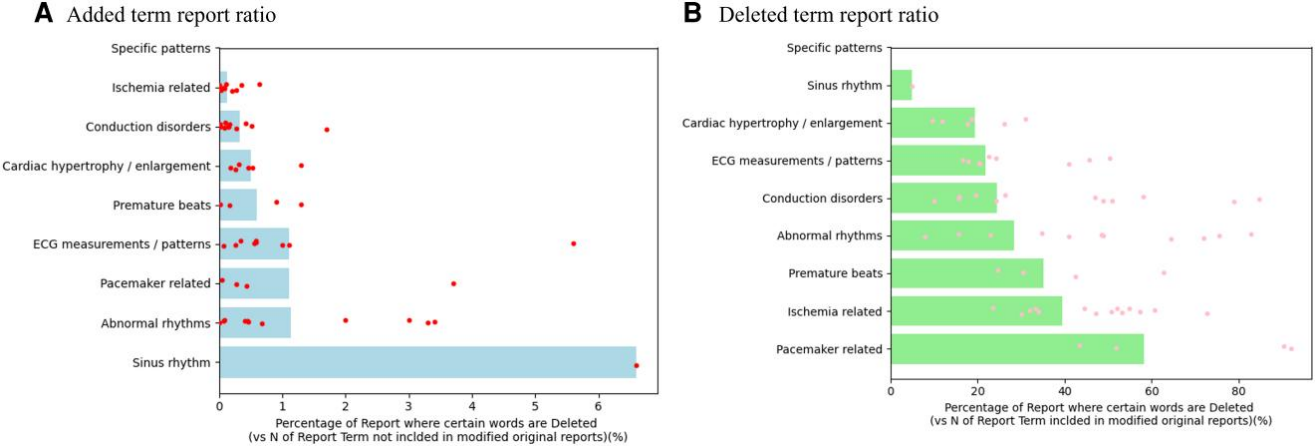
The added term report ratio (*A*) and the deleted term report ratio (*B*) for different categories of ECG terminology after physician review. (*A*) The blue horizontal bars represent the average percentage of reports within each terminology category where terms were newly added by the physician. The red dots represent the added term report ratio for individual ECG terms within each category (Refer Supplementary material online, *Table S4* in detail). (*B*) The green horizontal bars represent the average percentage of reports within each terminology category where terms were deleted by the physician. The pink dots represent the deleted term report ratio for individual ECG terms within each category (refer Supplementary material online, *Table S4* in detail).

Substantial differences in both the added report ratio and the deleted report ratio were observed across the nine categories of ECG-related terms (*Figure 4*). Notably, words belonging to the ‘Specific disease patterns’ category and ‘Ischaemia related’ category were rarely added to reports after physician review (0.03% and 0.13%, respectively). In contrast, ECG terms within the ‘ECG measurements/patterns’ category were added in 1.1% of the reports where they were initially absent (*P* < 0.001) (*Figure 4A* and *Table 3*). While words within the ‘ECG measurements/patterns’ category were deleted in 21.9% of the reports where they initially appeared, the modification rate was significantly higher for terms in the ‘Pacemaker related’ category, reaching 58.2% (*P* < 0.001) (*Figure 4B* and *Table 3*). Analysis of modification rates across nine categories was also performed based on physician groups (the eight physicians from whom ECGs were randomly sampled and all other physicians). While no large difference was observed between the two groups for added term reports ratio, a large difference was demonstrated for deleted term report ratio, particularly in the ‘Pacemaker related’ category (76.0% in the eight-physician group vs. 51.3% in the remaining group) (see Supplementary material online, *Figure S3*).

**Table 3:** Changes in ECG terminology by category after physician review

Terminology category	Number of terms	Number of reports where the term is absent	Total term frequency/*N* of original reports^a^	Total term frequency/*N* of final reports^a^	Term added (vs. originally absent)	Term removed (vs. originally present)
Sinus rhythm	1	24 526	25 592/25 363	25 911/25 737	1619 (6.6%)	1245 (4.9%)
ECG measurements/patterns	9	430 802	18 275/18 199	18 992/18 941	4721 (1.1%)	3979 (21.9%)
Abnormal rhythms	12	581 113	18 011/17 555	19 428/19 173	6589 (1.13%)	4971 (28.3%)
Pre-mature beats	4	192 375	7814/7181	6089/5817	1158 (0.6%)	2522 (35.1%)
Conduction disorders	14	682 216	16 545/16 230	14 558/14 441	2183 (0.32%)	3972 (24.5%)
Ischaemia related	14	679 947	19 386/18 499	12 335/12 078	869 (0.13%)	7290 (39.4%)
Cardiac hypertrophy/enlargement	6	290 586	8748/8748	8503/8498	1446 (0.5%)	1696 (19.4%)
Pacemaker related	4	198 423	1257/1133	2720/2669	2195 (1.11%)	659 (58.2%)
Specific disease patterns	5	247 632	1873/1813	1245/1200	69 (0.03%)	682 (37.6%)

^a^ECG term occasionally appears multiple times in a single report. ‘*N* of original (or final) reports’ is the number of reports in which that ECG term appeared, and ‘total term frequency’ is the total count of all occurrences of the term in those reports.

In a secondary analysis, physicians with a relatively limited experience were more likely to accept the automated ECG report, while there was an increasing trend in the modification rate with increasing physician experience (24.7% (<15-year experience) vs. 41.5% (16–23 year experience) and 28.4% (over 36-year experience), *P* < 0.0001) (see Supplementary material online, *Table S6*). Furthermore, the modification rate was observed to be higher for older Marquette™ 12SL ECG analysis programmes. Additionally, there were no significant modification rates based on the location where the ECGs were performed (from 32.1% in general wards to 33.8% in intensive care units, Supplementary material online, *Table S2*).

## Discussion

This work offers a systematic, real-world evaluation of how clinicians interact with automatically generated ECG interpretations. Our aim was to examine how physicians interpret, modify, or accept these outputs in everyday practice, an essential but underexplored aspect of algorithm integration in clinical workflows. In our large-scale retrospective analysis of 159 630 ECGs finalized, we found that about 30% of automated ECG interpretations were modified by physicians. These modifications were significantly more likely in ECGs obtained during off-hours, in patients with abnormal heart rates and prolonged QRS durations, scenarios that often-required nuanced clinical interpretation. Terms such as ‘sinus rhythm’ were both frequently added and removed, reflecting nuanced human judgment in ambiguous rhythm contexts.

Physician behaviour can be varied by experience level, with more seasoned clinicians (>36 years post-graduation) exhibiting higher modification rates than their less experienced counterparts, suggesting that experience may correlate with greater scrutiny or confidence in overriding the algorithm. And the temporal trends also revealed that modifications decreased over time, potentially due to iterative improvements in the underlying ECG algorithm; reports generated with newer versions of the Marquette™ 12SL system had significantly lower modification rates than earlier ones (18.4% in 2023 vs. 42.2% in 2011–12). Collectively, these findings highlight that physicians do not simply correct erroneous outputs. They actively reassess and refine automated outputs, often de-emphasizing vague or misleading terms while reinstating clinically salient ones.^11^ This selective editing process reflects deep contextual reasoning, shaped by patient presentation, institutional norms, and the evolving accuracy of algorithmic tools. These are critical elements that future AI models must incorporate in order to serve as trustworthy diagnostic partners.

Our term-level analysis further revealed that physicians frequently removed ischaemia-specific ECG terms such as ‘inferior infarct’ and ‘anterior infarct’ (deleted in 32.0% and 44.6% of cases, respectively) while preferentially adding diagnostically specific terms such as ‘prolonged QT interval’ and ‘electronic ventricular pacemaker’. The frequent physician addition of ‘prolonged QT interval’ suggests systematic under-detection by the algorithm, which may stem from conservative QT thresholding, algorithmic suppression due to coexisting waveform abnormalities or measurement inaccuracies, limitations previously reported in commercial ECG systems.^10^ These modifications were not uniformly distributed but exhibited distinct patterns across ECG term categories, physician experience levels, and algorithm versions. For example, ECG terms related to pacemakers and ischaemia were disproportionately deleted by physicians, up to 58.2% and 39.4% of the time, suggesting that automated interpretations in these domains frequently overcall or misclassified. Conversely, terms reflecting precise measurements or rhythm descriptors, such as those in the ‘ECG measurements/patterns’ and ‘Bradycardia/Tachycardia’ categories, were more often added during physician review, indicating areas where the algorithm may be overly conservative or nonspecific. Some terms like ‘suspect unspecified pacemaker failure’ and ‘undetermined rhythm’ were removed in over 82% of cases when initially present. These terms are often nonspecific and clinically ambiguous, providing limited diagnostic value or actionable insight, which likely drives their high deletion rate.^4^

Previous research evaluating commercial automated ECG interpretation systems has consistently highlighted their diagnostic limitations, particularly in real-world clinical settings.^1,4^ These rule-based systems rely on fixed thresholds and pre-defined criteria to detect abnormalities, which can result in high specificity for well-defined patterns but reduced sensitivity for more subtle or atypical presentations. For instance, one study reported that a commercial ECG algorithm identified only 63% of true ST-elevation myocardial infarctions, with even lower performance for non-ST elevation acute coronary syndromes, thus posing a risk in emergent care settings where timely diagnosis is critical.^4^ Similarly, when directly compared with physician interpretations, conventional ECG programmes generally demonstrate lower accuracy, especially against cardiologists.^12^ More recent analyses show that computer algorithms tend to have similar sensitivity as physicians for obvious findings but lack the nuanced pattern recognition of an experienced reader, resulting in more frequent errors of interpretation. In one report, the median diagnostic accuracy of several popular ECG machines was about 6% lower than that of cardiologists.^13^ Physicians are better at integrating clinical context and subtle waveform clues, whereas the software may misclassify artefact as arrhythmia or miss atypical presentations. Notably, having the computer’s preliminary read can influence clinicians, a correct machine interpretation can aid physician diagnosis, but an incorrect one can bias the human reader towards a wrong conclusion.^1^ This ‘automation bias’ is a concern, particularly for less experienced doctors.

Recently, deep learning models have emerged as a powerful alternative, capable of surpassing traditional ECG algorithms in both accuracy and breadth of interpretation across a range of cardio-metabolic disorders. Deep learning has shown the capability to interpret ECGs across a broad range of cardiac conditions, including arrhythmia classification,^14^ detection of structural heart disease,^15,16^ and inference of hidden biomarkers associated with conditions like hyperkalemia, obstructive coronary artery disease, atrial fibrillation during sinus rhythm, and even heart age^17–22^; furthermore, real-world implementation studies have shown that AI-generated ECG reports more closely match final physician assessments and require fewer major edits than those produced by standard commercial software like GE 12SL, suggesting immediate clinical utility.^23^ These findings suggest that deep learning approaches not only match expert-level detection capabilities but also reduce the interpretive burden on clinicians. As these models continue to be validated across broader populations and integrated into clinical workflows, they hold the potential to enhance the accuracy, efficiency, and trustworthiness of automated ECG interpretation far beyond what legacy systems can offer.^24^

### Limitations

This study has several limitations. First, the analysis was conducted at a single academic medical centre using a specific commercial ECG interpretation system (Marquette™ 12SL by GE Healthcare), which may limit the generalizability of our findings to other institutions or ECG software. A significant limitation of this study is the highly skewed distribution of ECG interpretations among physicians, where a small number of physicians read a disproportionately large volume of ECGs. Although random sampling was performed, more than half of the data were still interpreted by eight physicians. Furthermore, the removal of a substantial portion of the interpretation results from eight physicians through sampling could lead to potential selection bias in various downstream analyses. Second, while ECG terms were categorized systematically by experienced clinicians, the classification process inherently involved subjective judgement. This approach may not fully capture nuanced semantic or contextual modification, particularly those extending beyond direct keyword-level analysis. Third, while our dataset spans more than a decade, we were unable to individually account for temporal factors such as physician turnover, evolving institutional practices, or associations with patients’ downstream clinical outcomes due to limitations in the available data.

## Conclusion

This work offers a large-scale, real-world assessment of how clinicians interact with algorithm-generated ECG reports. Our findings reflect physicians’ clinical reasoning, contextual interpretation, and judgment in adapting automated outputs to real-world scenarios. The consistent patterns of modification we observed underscore the limitations of current rule-based systems and the indispensable role of physician oversight. As deep learning models increasingly demonstrate enhanced accuracy and broader diagnostic capability, future research should focus on validating these systems in diverse clinical environments and integrating them into workflows in a way that complements clinical reasoning. By identifying ECG features most prone to algorithmic errors or omissions, our findings provide actionable insights for guiding the development of future AI models and refining clinical training strategies, supporting a shift from static, rules-based outputs towards adaptive, context-aware algorithms that align more closely with real-world diagnostic practice.

## Supplementary Material

ztaf119_Supplementary_Data

## Data Availability

The data underlying this article cannot be shared publicly due to the privacy of individuals that participated in the study. The data will be shared on reasonable request to the corresponding author.
