# Supplementary material for: Factors associated with physician modifications to automated ECG interpretations
Source: Eur Heart J Digit Health. 2025 Nov 8;7(1):ztaf119. doi: 10.1093/ehjdh/ztaf119 (PMC12821064; doi:10.1093/ehjdh/ztaf119)
Supplement: ztaf119_Supplementary_Data [file ztaf119_supplementary_data.zip › ECG_interpretation_Supple_R4_Clear_v1.0.docx]

**Supplemental Materials (Figures)**

**Supplemental Figure 1: Distribution of ECG Reviews and Modifications by Physicians.**

**
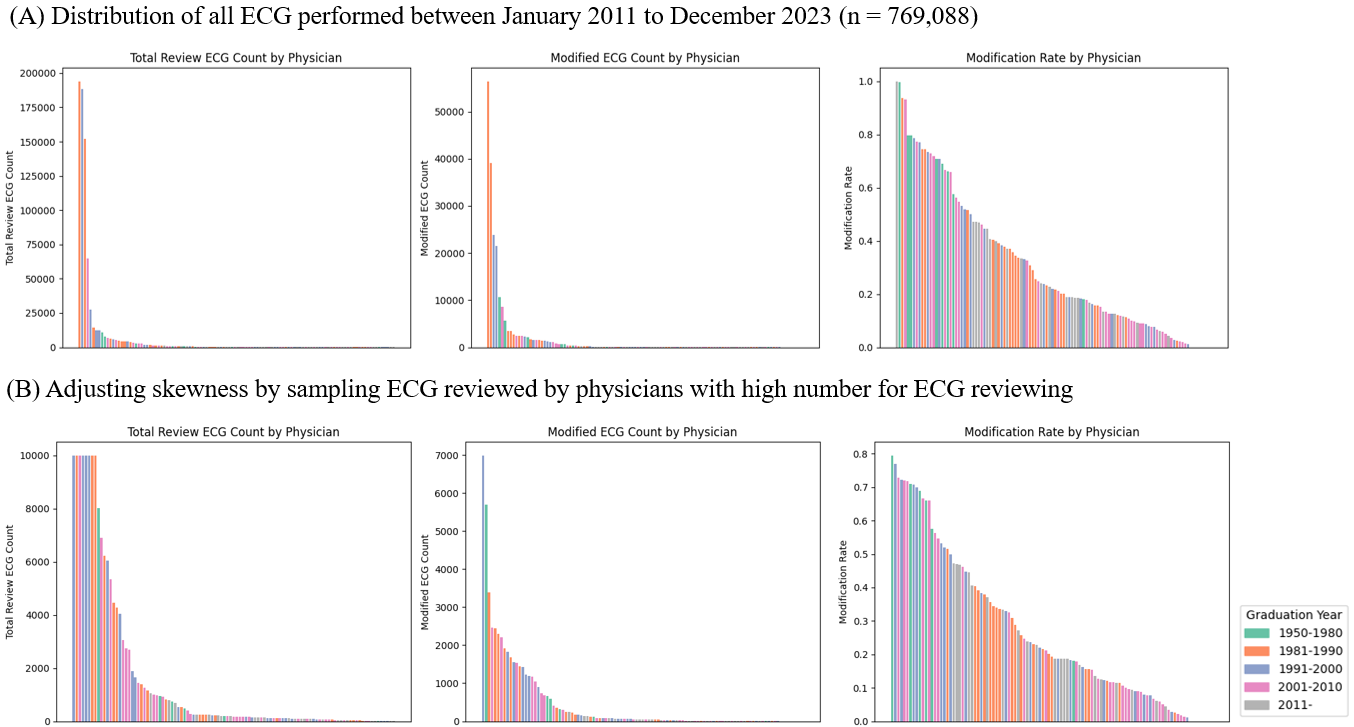
**

(A) Distribution of all ECGs reviewed and modified by physicians between January 2011 and December 2023 (n = 769,088). The left panel shows the total number of reviewed ECGs per physician, the middle panel shows the number of modified ECGs per physician, and the right panel demonstrates the modification rate per physician.

(B) Distribution of all ECGs reviewed and modified by physicians after sampling ECGs reviewed by physicians with a high number of ECG reviews to address significant skewness (n = 159,630). The panels represent the same metrics as in (A).

**Supplemental Figure 2: The Study flowchart**

**
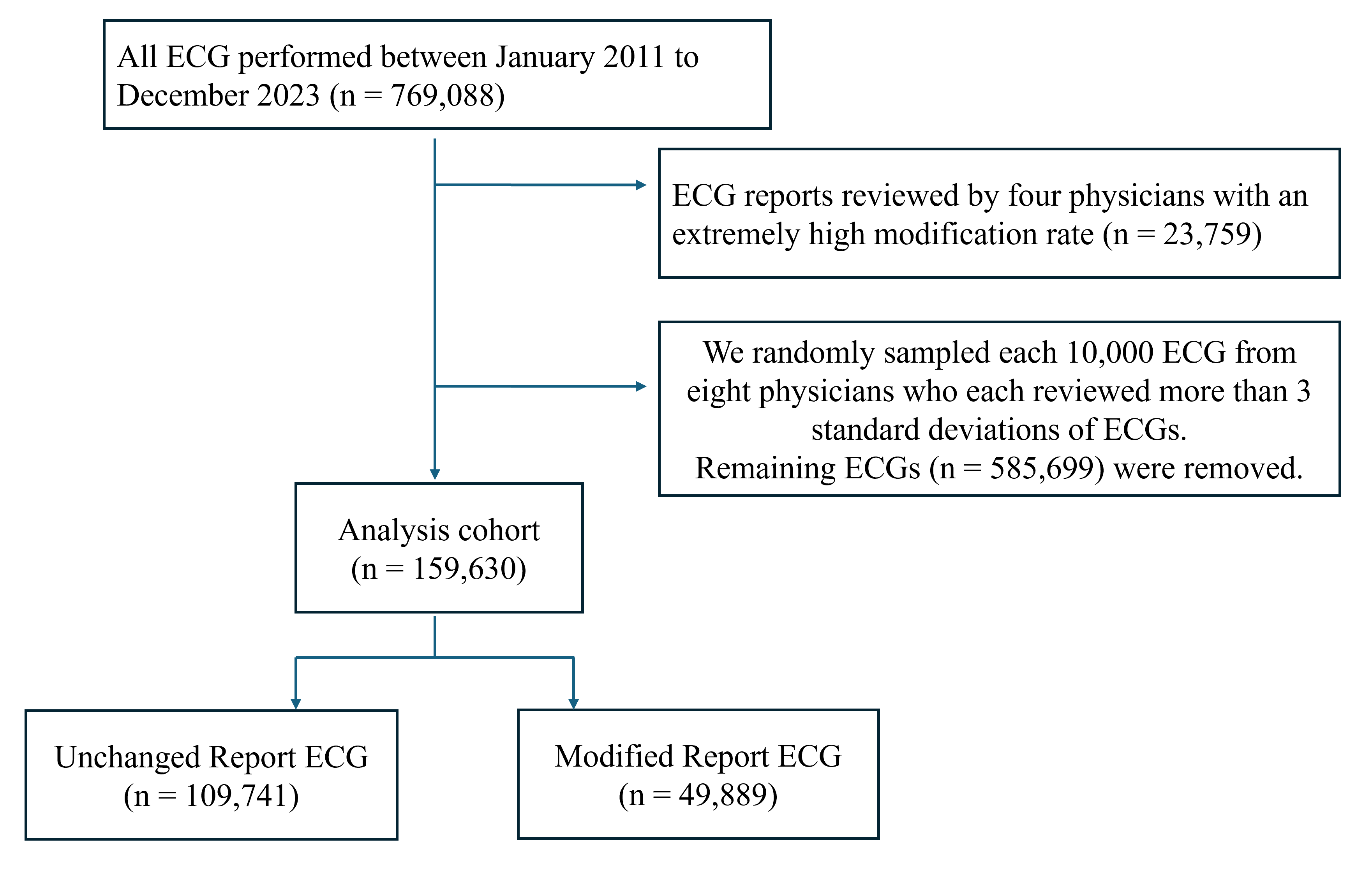
**

**Supplemental Materials (Tables)**

**Supplemental Table 1**: ECG terminology list and category

| Category | ECG Term |
| --- | --- |
| Sinus rhythm | sinus rhythm |
| ECG measurements / patterns | left axis deviation |
|  | prolonged qt interval |
|  | low voltage qrs |
|  | st elevation |
|  | nonspecific st abnormality |
|  | right axis deviation |
|  | early repolarization |
|  | indeterminate axis |
|  | st depression |
| Abnormal rhythms | sinus tachycardia |
|  | sinus bradycardia |
|  | atrial fibrillation |
|  | junctional rhythm |
|  | atrial flutter |
|  | supraventricular tachycardia |
|  | wide qrs tachycardia |
|  | idioventricular rhythm |
|  | narrow qrs tachycardia |
|  | ventricular tachycardia  ectopic atrial rhythm |
| Premature beats | premature ventricular complexes |
|  | premature atrial complexes |
|  | fusion complexes |
|  | supraventricular complexes |
| Conduction disorders | right bundle branch block |
|  | 1st degree av block |
|  | nonspecific intraventricular block |
|  | left anterior fascicular block |
|  | left bundle branch block |
|  | wide qrs rhythm |
|  | bifascicular block |
|  | aberrant conduction |
|  | left posterior fascicular block |
|  | 2nd degree av block |
|  | complete heart block |
|  | wpw pattern |
|  | av dissociation |
|  | complete av block |
| Ischemia related | inferior infarct |
|  | anterior infarct |
|  | septal infarct |
|  | acute mi |
|  | lateral infarct |
|  | anterolateral infarct |
|  | anteroseptal infarct |
|  | stemi |
|  | posterior infarct |
|  | lateral injury pattern |
|  | inferior injury pattern |
|  | inferolateral injury pattern |
|  | anterior injury pattern |
|  | anterolateral injury pattern |
| Cardiac hypertrophy / enlargement | left ventricular hypertrophy |
|  | left atrial enlargement |
|  | right ventricular hypertrophy |
|  | right atrial enlargement |
|  | biventricular hypertrophy |
|  | biatrial enlargement |
| Pacemaker related | electronic atrial pacemaker |
|  | electronic ventricular pacemaker |
|  | suspect unspecified pacemaker failure |
|  | atrial tracking ventricular paced rhythm |
| Specific patterns | undetermined rhythm  digitalis effect |
|  | pulmonary disease pattern |
|  | acute pericarditis |
|  | dextrocardia |
|  | brugada pattern |

**Supplemental Table 2**: Modification rate by ECG testing department

|  | | | GE Marquette™ 12SL ECG analysis program | | | | | | |
| --- | --- | --- | --- | --- | --- | --- | --- | --- | --- |
| Location Category | Modification Rate | Total Count | version 16 | version 18 | version 19 | version 20 | version 21 | version 22 | version 23 |
| Outpatient Clinic | 32.5% | 24023 | 226 | 0 | 43 | 3038 | 1855 | 10537 | 8324 |
| CCU / ICU | 33.8% | 34933 | 0 | 0 | 1 | 10278 | 3130 | 7274 | 14250 |
| Emergency Department | 33.7% | 23335 | 0 | 0 | 4889 | 8127 | 3939 | 346 | 6034 |
| General Ward | 32.1% | 44303 | 1 | 0 | 5 | 13479 | 6139 | 10077 | 14602 |

33,036 ECGs have missing or unknown information regarding the location where they were performed. CCU: cardiac care units. ICU: Intensive care unit

**Supplemental Table 3**: Comparison of ECG Terms frequency in Unchanged and Modified reports

|  | Unchanged Report (N = 109,741) | | Modified Report (N = 49,889) | |
| --- | --- | --- | --- | --- |
| Term | Original report with Term (Same report) | Original report with Term (Modified report) | Ratio (Same report) | Ratio (Modified report) |
| Sinus rhythm | | | | |
| sinus rhythm | 67775 | 25363 | 61.76% | 50.84% |
| ECG measurements / patterns | | | | |
| left axis deviation | 10984 | 6347 | 10.01% | 12.72% |
| prolonged qt interval | 8550 | 4313 | 7.79% | 8.65% |
| low voltage qrs | 6703 | 3361 | 6.11% | 6.74% |
| nonspecific st abnormality | 2514 | 1140 | 2.29% | 2.29% |
| early repolarization | 936 | 577 | 0.85% | 1.16% |
| st elevation | 875 | 1208 | 0.8% | 2.42% |
| right axis deviation | 752 | 873 | 0.69% | 1.75% |
| st depression | 184 | 187 | 0.17% | 0.37% |
| indeterminate axis | 160 | 193 | 0.15% | 0.39% |
| Abnormal rhythms | | | | |
| sinus bradycardia | 11837 | 4019 | 10.79% | 8.06% |
| sinus tachycardia | 10325 | 5179 | 9.41% | 10.38% |
| atrial fibrillation | 8000 | 3923 | 7.29% | 7.86% |
| atrial flutter | 1208 | 961 | 1.1% | 1.93% |
| junctional rhythm | 460 | 1286 | 0.42% | 2.58% |
| undetermined rhythm | 219 | 1489 | 0.2% | 2.98% |
| supraventricular tachycardia | 207 | 268 | 0.19% | 0.54% |
| ectopic atrial rhythm | 181 | 186 | 0.16% | 0.37% |
| wide qrs tachycardia | 86 | 186 | 0.08% | 0.37% |
| idioventricular rhythm | 23 | 33 | 0.02% | 0.07% |
| narrow qrs tachycardia | 2 | 25 | 0.0% | 0.05% |
| ventricular tachycardia | 0 | 0 | 0 | 0.0% |
| Premature beats | | | | |
| premature ventricular complexes | 4994 | 3201 | 4.55% | 6.42% |
| premature atrial complexes | 4114 | 2037 | 3.75% | 4.08% |
| supraventricular complexes | 1357 | 851 | 1.24% | 1.71% |
| fusion complexes | 805 | 1092 | 0.73% | 2.19% |
| Conduction disorders | | | | |
| right bundle branch block | 11287 | 5232 | 10.29% | 10.49% |
| 1st degree av block | 7618 | 3201 | 6.94% | 6.42% |
| left anterior fascicular block | 3867 | 1712 | 3.52% | 3.43% |
| left bundle branch block | 3435 | 1473 | 3.13% | 2.95% |
| nonspecific intraventricular block | 1789 | 1945 | 1.63% | 3.9% |
| bifascicular block | 1614 | 718 | 1.47% | 1.44% |
| aberrant conduction | 647 | 538 | 0.59% | 1.08% |
| left posterior fascicular block | 369 | 227 | 0.34% | 0.46% |
| wide qrs rhythm | 245 | 853 | 0.22% | 1.71% |
| 2nd degree av block | 150 | 224 | 0.14% | 0.45% |
| complete heart block | 65 | 81 | 0.06% | 0.16% |
| wpw pattern | 17 | 26 | 0.02% | 0.05% |
| av dissociation | 0 | 0 | N/A | 0.0% |
| complete av block | 0 | 0 | N/A | 0.0% |
| Ischemia related | | | | |
| inferior infarct | 8169 | 6011 | 7.44% | 12.05% |
| anterior infarct | 4706 | 3077 | 4.29% | 6.17% |
| septal infarct | 4266 | 2746 | 3.89% | 5.5% |
| anteroseptal infarct | 1854 | 1189 | 1.69% | 2.38% |
| anterolateral infarct | 1825 | 1295 | 1.66% | 2.6% |
| lateral infarct | 1402 | 1398 | 1.28% | 2.8% |
| acute mi | 823 | 1428 | 0.75% | 2.86% |
| stemi | 510 | 744 | 0.46% | 1.49% |
| posterior infarct | 330 | 286 | 0.3% | 0.57% |
| lateral injury pattern | 65 | 136 | 0.06% | 0.27% |
| inferior injury pattern | 41 | 104 | 0.04% | 0.21% |
| inferolateral injury pattern | 21 | 32 | 0.02% | 0.06% |
| anterolateral injury pattern | 19 | 25 | 0.02% | 0.05% |
| anterior injury pattern | 6 | 28 | 0.01% | 0.06% |
| Cardiac hypertrophy / enlargement | | | | |
| left atrial enlargement | 5425 | 2407 | 4.94% | 4.82% |
| left ventricular hypertrophy | 5125 | 3355 | 4.67% | 6.72% |
| right ventricular hypertrophy | 1136 | 1710 | 1.04% | 3.43% |
| right atrial enlargement | 674 | 561 | 0.61% | 1.12% |
| biatrial enlargement | 399 | 188 | 0.36% | 0.38% |
| biventricular hypertrophy | 248 | 527 | 0.23% | 1.06% |
| Pacemaker related | | | | |
| electronic atrial pacemaker | 449 | 458 | 0.41% | 0.92% |
| electronic ventricular pacemaker | 114 | 395 | 0.1% | 0.79% |
| atrial tracking ventricular paced rhythm | 36 | 126 | 0.03% | 0.25% |
| suspect unspecified pacemaker failure | 12 | 154 | 0.01% | 0.31% |
| Specific disease patterns | | | | |
| digitalis effect | 1446 | 1036 | 1.32% | 2.08% |
| pulmonary disease pattern | 916 | 608 | 0.83% | 1.22% |
| acute pericarditis | 69 | 106 | 0.06% | 0.21% |
| brugada pattern | 8 | 6 | 0.01% | 0.01% |
| dextrocardia | 6 | 57 | 0.01% | 0.11% |

**Supplemental Table 4**: Modification patterns in all specific ECG terminology by physician review

| Term | Report - Term Present -Original (n) | Ratio | Report - Term Absent (n) | Report - Term Present -After Review (n) | Total Term Count (Before / After Review) | Report - Term Added (n) | Report - Term Deleted (n) |
| --- | --- | --- | --- | --- | --- | --- | --- |
| Sinus rhythm | | | | | | | |
| sinus rhythm | 25363 | 50.84% | 24526 | 25737 | 25592 / 25911 (1.2%) | 1619 (6.6%) | 1245 (4.9%) |
| ECG measurements / patterns | | | | | | | |
| left axis deviation | 6347 | 12.72% | 43542 | 5473 | 6347 / 5478 (-13.69%) | 254 (0.58%) | 1128 (17.8%) |
| prolonged qt interval | 4313 | 8.65% | 45576 | 5906 | 4313 / 5906 (36.9%) | 2565 (5.6%) | 972 (22.5%) |
| low voltage qrs | 3361 | 6.74% | 46528 | 2944 | 3361 / 2947 (-12.32%) | 271 (0.58%) | 688 (20.5%) |
| st elevation | 1208 | 2.42% | 48681 | 1217 | 1284 / 1255 (-2.26%) | 503 (1.0%) | 494 (40.9%) |
| nonspecific st abnormality | 1140 | 2.29% | 48749 | 1179 | 1140 / 1179 (3.4%) | 270 (0.55%) | 231 (20.3%) |
| right axis deviation | 873 | 1.75% | 49016 | 896 | 873 / 898 (2.9%) | 167 (0.34%) | 144 (16.5%) |
| early repolarization | 577 | 1.16% | 49312 | 966 | 577 / 969 (67.9%) | 529 (1.1%) | 140 (24.3%) |
| indeterminate axis | 193 | 0.39% | 49696 | 139 | 193 / 139 (-27.98%) | 34 (0.07%) | 88 (45.6%) |
| st depression | 187 | 0.37% | 49702 | 221 | 187 / 221 (18.2%) | 128 (0.26%) | 94 (50.3%) |
| Abnormal rhythms | | | | | | | |
| sinus tachycardia | 5179 | 10.38% | 44710 | 5719 | 5179 / 5724 (10.5%) | 1348 (3.0%) | 808 (15.6%) |
| sinus bradycardia | 4019 | 8.06% | 45870 | 5278 | 4019 / 5290 (31.6%) | 1582 (3.4%) | 323 (8.0%) |
| atrial fibrillation | 3923 | 7.86% | 45966 | 4548 | 4141 / 4675 (12.9%) | 1529 (3.3%) | 904 (23.0%) |
| undetermined rhythm | 1489 | 2.98% | 48400 | 472 | 1563 / 474 (-69.67%) | 217 (0.45%) | 1234 (82.9%) |
| junctional rhythm | 1286 | 2.58% | 48603 | 541 | 1370 / 563 (-58.91%) | 226 (0.46%) | 971 (75.5%) |
| atrial flutter | 961 | 1.93% | 48928 | 1565 | 1019 / 1618 (58.8%) | 997 (2.0%) | 393 (40.9%) |
| supraventricular tachycardia | 268 | 0.54% | 49621 | 507 | 268 / 529 (97.4%) | 332 (0.67%) | 93 (34.7%) |
| wide qrs tachycardia | 186 | 0.37% | 49703 | 112 | 195 / 112 (-42.56%) | 46 (0.09%) | 120 (64.5%) |
| ectopic atrial rhythm | 186 | 0.37% | 49703 | 299 | 195 / 307 (57.4%) | 204 (0.41%) | 91 (48.9%) |
| idioventricular rhythm | 33 | 0.07% | 49856 | 53 | 37 / 57 (54.1%) | 36 (0.07%) | 16 (48.5%) |
| narrow qrs tachycardia | 25 | 0.05% | 49864 | 12 | 25 / 12 (-52.0%) | 5 (0.01%) | 18 (72.0%) |
| ventricular tachycardia | 0 | 0.0% | 49889 | 67 | 0 / 67 | 67 | 0 |
| Premature beats | | | | | | | |
| premature ventricular complexes | 3201 | 6.42% | 46688 | 2648 | 3492 / 2792 (-20.05%) | 419 (0.9%) | 972 (30.4%) |
| premature atrial complexes | 2037 | 4.08% | 47852 | 2179 | 2203 / 2258 (2.5%) | 645 (1.3%) | 503 (24.7%) |
| fusion complexes | 1092 | 2.19% | 48797 | 489 | 1194 / 510 (-57.29%) | 82 (0.17%) | 685 (62.7%) |
| supraventricular complexes | 851 | 1.71% | 49038 | 501 | 925 / 529 (-42.81%) | 12 (0.02%) | 362 (42.5%) |
| Conduction disorders | | | | | | | |
| right bundle branch block | 5232 | 10.49% | 44657 | 5486 | 5332 / 5556 (4.2%) | 777 (1.7%) | 523 (10.0%) |
| 1st degree av block | 3201 | 6.42% | 46688 | 3233 | 3201 / 3234 (-9.59%) | 536 (0.42%) | 504 (15.7%) |
| nonspecific intraventricular block | 1945 | 3.9% | 47944 | 1062 | 1958 / 1063 (-45.71%) | 69 (0.14%) | 952 (48.9%) |
| left anterior fascicular block | 1712 | 3.43% | 48177 | 1570 | 1736 / 1578 (-9.1%) | 131 (0.27%) | 273 (15.9%) |
| left bundle branch block | 1473 | 2.95% | 48416 | 1336 | 1519 / 1353 (-10.93%) | 251 (0.52%) | 388 (26.3%) |
| wide qrs rhythm | 853 | 1.71% | 49036 | 221 | 907 / 224 (-75.3%) | 41 (0.08%) | 673 (78.9%) |
| bifascicular block | 718 | 1.44% | 49171 | 625 | 718 / 625 (-12.95%) | 47 (0.1%) | 140 (19.5%) |
| aberrant conduction | 538 | 1.08% | 49351 | 305 | 601 / 313 (-47.92%) | 41 (0.08%) | 274 (50.9%) |
| left posterior fascicular block | 227 | 0.46% | 49662 | 183 | 230 / 183 (-20.43%) | 11 (0.02%) | 55 (24.2%) |
| 2nd degree av block | 224 | 0.45% | 49665 | 223 | 236 / 230 (-24.58%) | 129 (0.16%) | 130 (58.0%) |
| complete heart block | 81 | 0.16% | 49808 | 119 | 81 / 120 (48.1%) | 76 (0.15%) | 38 (46.9%) |
| wpw pattern | 26 | 0.05% | 49863 | 16 | 26 / 16 (-38.46%) | 12 (0.02%) | 22 (84.6%) |
| av dissociation | 0 | 0.0% | 49889 | 46 | 0 / 47 | 46 | 0 |
| complete av block | 0 | 0.0% | 49889 | 16 | 0 / 16 | 16 | 0 |
| Ischemia related | | | | | | | |
| inferior infarct | 6011 | 12.05% | 43878 | 4361 | 6224 / 4463 (-28.29%) | 275 (0.63%) | 1925 (32.0%) |
| anterior infarct | 3077 | 6.17% | 46812 | 1874 | 3123 / 1893 (-39.39%) | 168 (0.36%) | 1371 (44.6%) |
| septal infarct | 2746 | 5.5% | 47143 | 1909 | 2841 / 1949 (-31.4%) | 96 (0.2%) | 933 (34.0%) |
| acute mi | 1428 | 2.86% | 48461 | 415 | 1428 / 415 (-70.94%) | 25 (0.05%) | 1038 (72.7%) |
| lateral infarct | 1398 | 2.8% | 48491 | 795 | 1442 / 803 (-44.31%) | 55 (0.11%) | 658 (47.1%) |
| anterolateral infarct | 1295 | 2.6% | 48594 | 944 | 1321 / 954 (-27.78%) | 39 (0.08%) | 390 (30.1%) |
| anteroseptal infarct | 1189 | 2.38% | 48700 | 1039 | 1225 / 1063 (-13.22%) | 131 (0.27%) | 281 (23.6%) |
| stemi | 744 | 1.49% | 49145 | 332 | 1165 / 384 (-67.04%) | 14 (0.03%) | 426 (57.3%) |
| posterior infarct | 286 | 0.57% | 49603 | 237 | 292 / 238 (-18.49%) | 46 (0.09%) | 95 (33.2%) |
| lateral injury pattern | 136 | 0.27% | 49753 | 69 | 136 / 69 (-49.26%) | 2 (0.0%) | 69 (50.7%) |
| inferior injury pattern | 104 | 0.21% | 49785 | 52 | 104 / 53 (-49.04%) | 5 (0.01%) | 57 (54.8%) |
| inferolateral injury pattern | 32 | 0.06% | 49857 | 19 | 32 / 19 (-40.62%) | 4 (0.01%) | 17 (53.1%) |
| anterior injury pattern | 28 | 0.06% | 49861 | 15 | 28 / 15 (-46.43%) | 4 (0.01%) | 17 (60.7%) |
| anterolateral injury pattern | 25 | 0.05% | 49864 | 17 | 25 / 17 (-32.0%) | 5 (0.01%) | 13 (52.0%) |
| Cardiac hypertrophy / enlargement | | | | | | | |
| left ventricular hypertrophy | 3355 | 6.72% | 46534 | 2944 | 3355 / 2944 (-12.25%) | 214 (0.46%) | 625 (18.6%) |
| left atrial enlargement | 2407 | 4.82% | 47482 | 2733 | 2407 / 2735 (13.6%) | 610 (1.3%) | 284 (11.8%) |
| right ventricular hypertrophy | 1710 | 3.43% | 48179 | 1434 | 1710 / 1435 (-16.08%) | 256 (0.53%) | 532 (31.1%) |
| right atrial enlargement | 561 | 1.12% | 49328 | 613 | 561 / 614 (9.4%) | 151 (0.31%) | 99 (17.6%) |
| biventricular hypertrophy | 527 | 1.06% | 49362 | 516 | 527 / 517 (-1.9%) | 127 (0.26%) | 138 (26.2%) |
| biatrial enlargement | 188 | 0.38% | 49701 | 258 | 188 / 258 (37.2%) | 88 (0.18%) | 18 (9.6%) |
| Pacemaker related | | | | | | | |
| electronic atrial pacemaker | 458 | 0.92% | 49431 | 472 | 531 / 503 (-5.27%) | 213 (0.43%) | 199 (43.4%) |
| electronic ventricular pacemaker | 395 | 0.79% | 49494 | 2016 | 446 / 2036 (356.5%) | 1826 (3.7%) | 205 (51.9%) |
| suspect unspecified pacemaker failure | 154 | 0.31% | 49735 | 37 | 154 / 37 (-75.97%) | 22 (0.04%) | 139 (90.3%) |
| atrial tracking ventricular paced rhythm | 126 | 0.25% | 49763 | 144 | 126 / 144 (14.3%) | 134 (0.27%) | 116 (92.1%) |
| Specific disease patterns | | | | | | | |
| digitalis effect | 1036 | 2.08% | 48853 | 743 | 1096 / 787 (-28.19%) | 38 (0.08%) | 331 (31.9%) |
| pulmonary disease pattern | 608 | 1.22% | 49281 | 365 | 608 / 365 (-39.97%) | 4 (0.01%) | 247 (40.6%) |
| acute pericarditis | 106 | 0.21% | 49783 | 42 | 106 / 42 (-60.38%) | 6 (0.01%) | 70 (66.0%) |
| dextrocardia | 57 | 0.11% | 49832 | 40 | 57 / 41 (-28.07%) | 14 (0.03%) | 31 (54.4%) |
| brugada pattern | 6 | 0.01% | 49883 | 10 | 6 / 10 (66.7%) | 7 (0.01%) | 3 (50.0%) |

Supplemental Table 5: Categorized modification patterns in Top 3 ECG terminology by physician review

| Term | Terminology Grouping | Report - Term Present -Original (n) | Ratio | Report - Term Absent (n) | Report - Term Present -After Review (n) | Total Term Count (Before / After Review) | Report - Term Added (n) | Report - Term Deleted (n) |
| --- | --- | --- | --- | --- | --- | --- | --- | --- |
| Eight Physicians - Added Terms | | | | | | | | |
| prolonged qt interval | ECG measurements / patterns | 1520 | 7.7% | 18211 | 2628 | 1520 / 2628 (72.9%) | 1196 (6.6%) | 88 (5.8%) |
| atrial fibrillation | Abnormal rhythms | 1652 | 8.37% | 18079 | 1993 | 1664 / 1998 (20.1%) | 800 (4.4%) | 459 (27.8%) |
| electronic ventricular pacemaker | Pacemaker related | 30 | 0.15% | 19701 | 821 | 33 / 823 (2393.9%) | 806 (4.1%) | 15 (50.0%) |
| Eight Physicians - Deleted Terms | | | | | | | | |
| junctional rhythm | Abnormal rhythms | 604 | 3.06% | 19127 | 210 | 609 / 211 (-65.35%) | 94 (0.49%) | 488 (80.8%) |
| inferior infarct | Ischemia related | 2670 | 13.53% | 17061 | 1939 | 2732 / 1960 (-28.26%) | 47 (0.28%) | 778 (29.1%) |
| acute mi | Ischemia related | 716 | 3.63% | 19015 | 137 | 716 / 137 (-80.87%) | 11 (0.06%) | 590 (82.4%) |
| Other Physicians - Added Terms | | | | | | | | |
| prolonged qt interval | ECG measurements / patterns | 2793 | 9.26% | 27365 | 3278 | 2793 / 3278 (17.4%) | 1369 (5.0%) | 884 (31.7%) |
| sinus bradycardia | Abnormal rhythms | 2504 | 8.3% | 27654 | 3235 | 2504 / 3247 (29.7%) | 952 (3.4%) | 221 (8.8%) |
| electronic ventricular pacemaker | Pacemaker related | 365 | 1.21% | 29793 | 1195 | 413 / 1213 (193.7%) | 1020 (3.4%) | 190 (52.1%) |
| Other Physicians - Deleted Terms | | | | | | | | |
| prolonged qt interval | ECG measurements / patterns | 2793 | 9.26% | 27365 | 3278 | 2793 / 3278 (17.4%) | 1369 (5.0%) | 884 (31.7%) |
| inferior infarct | Ischemia related | 3341 | 11.08% | 26817 | 2422 | 3492 / 2503 (-28.32%) | 228 (0.85%) | 1147 (34.3%) |
| anterior infarct | Ischemia related | 1752.0 | 5.81% | 28406 | 950 | 1796 / 967 (-46.16%) | 130 (0.46%) | 932 (53.2%) |

**Supplemental Table 6**: Modification rate by physicians’ experience year

| Physicians Experience Year | ECG Count | Modification Rate |
| --- | --- | --- |
| <=15 | 53,786 | 24.7% |
| 16-23 | 25,550 | 41.5% |
| 24-30 | 36,917 | 29.8% |
| 31-36 | 29,314 | 37.6% |
| >36 | 14,063 | 28.4% |

Physicians experience year was adjusted to ensure that the number of electrocardiograms was evenly distributed across five quantiles.
