# Supplementary figures and images for: Factors associated with physician modifications to automated ECG interpretations
Source: Eur Heart J Digit Health. 2025 Nov 8;7(1):ztaf119. doi: 10.1093/ehjdh/ztaf119 (PMC12821064; doi:10.1093/ehjdh/ztaf119)

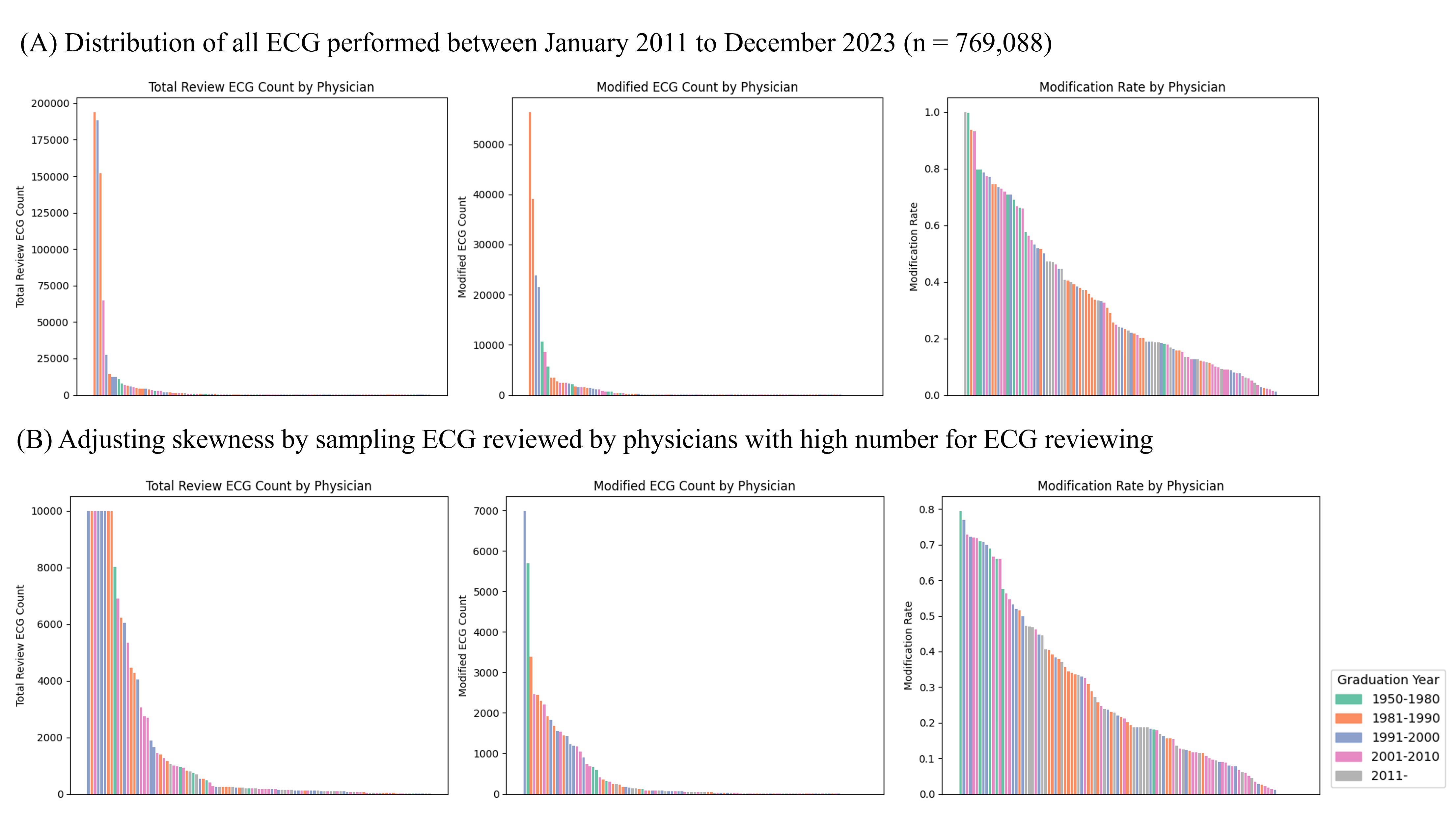

Supplement: ztaf119_Supplementary_Data [file ztaf119_supplementary_data.zip › Supplemental Figure 1.png]

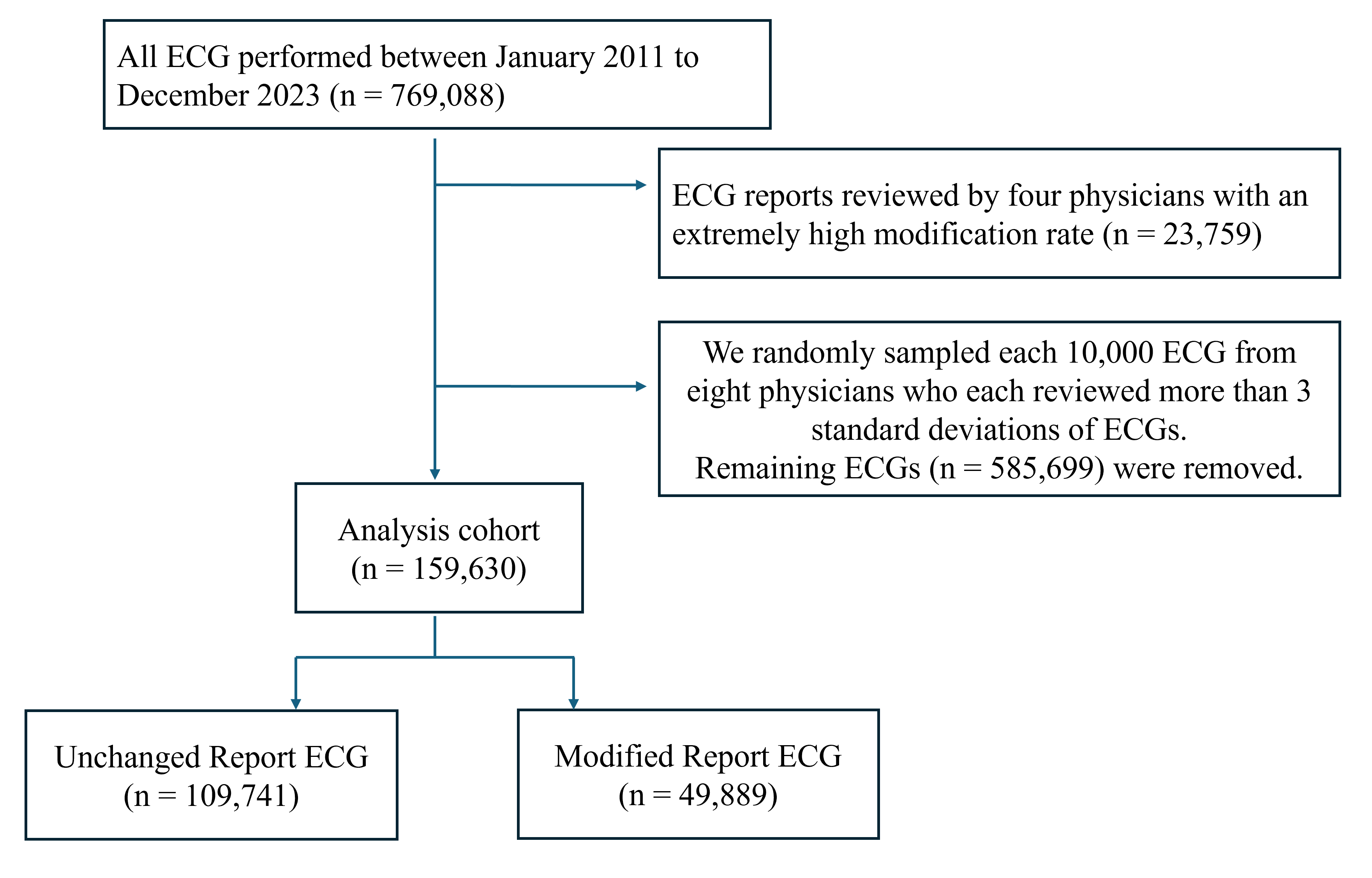

Supplement: ztaf119_Supplementary_Data [file ztaf119_supplementary_data.zip › Supplemental Figure 2.png]

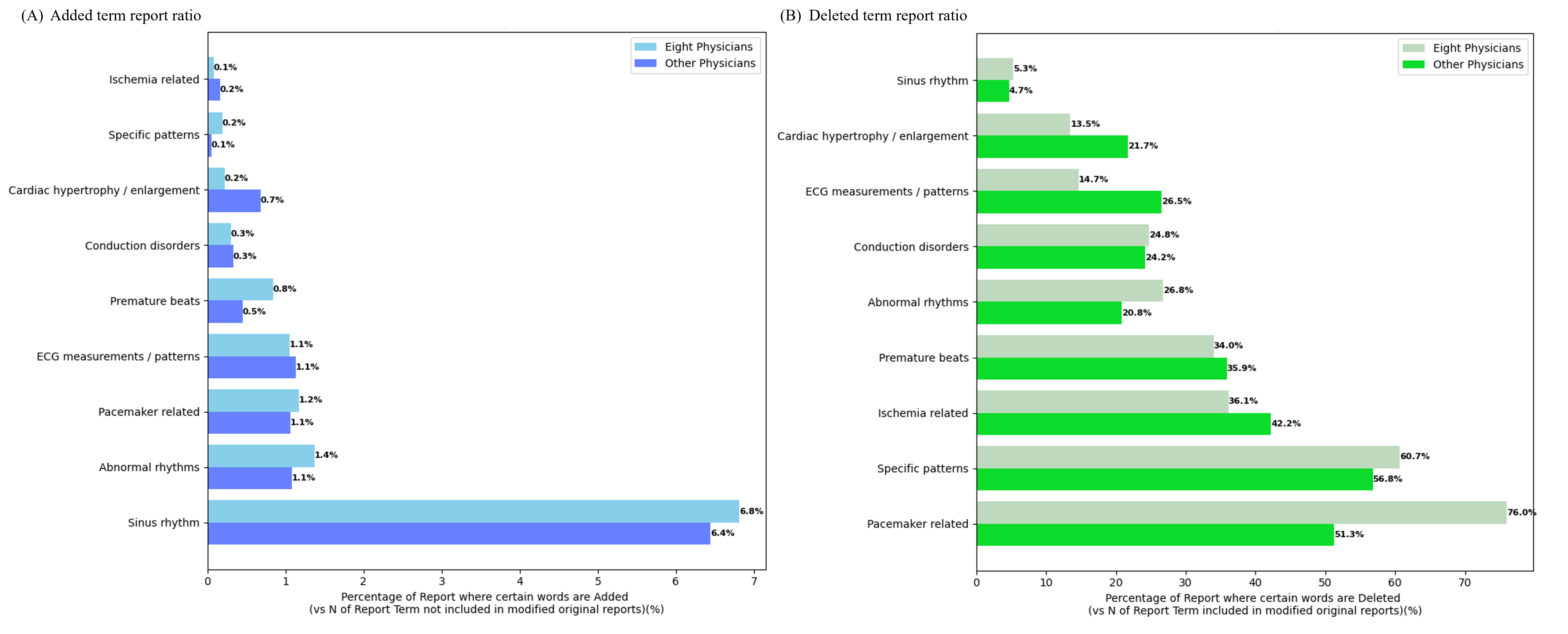

Supplement: ztaf119_Supplementary_Data [file ztaf119_supplementary_data.zip › Supplemental Figure 3.png]
